# Myasthenic Gravis Crisis in an 85-Year-Old Male Requiring Emergent Intubation and Plasmapheresis

**DOI:** 10.7759/cureus.80153

**Published:** 2025-03-06

**Authors:** Sara Yee, William R Rankin, Timothy Moore, Adam Brown, Douglas Rappaport, Wayne A Martini

**Affiliations:** 1 Emergency Medicine, Valleywise Health Medical Center, Phoenix, USA; 2 Emergency Medicine, Mayo Clinic Alix School of Medicine, Scottsdale, USA; 3 Emergency Medicine, Mayo Clinic Hospital, Phoenix, USA

**Keywords:** autoimmune disorder, bulbar muscle weakness, emergency intubation, immunomodulatory therapy, myasthenia gravis (mg), myasthenic crisis, neuromuscular junction, plasmapheresis, pyridostigmine withdrawal, respiratory failure

## Abstract

Myasthenia gravis (MG) is a chronic autoimmune neuromuscular disorder caused by autoantibodies targeting acetylcholine receptors at the neuromuscular junction, leading to impaired synaptic transmission and muscle weakness. Myasthenic crisis (MC) is a life-threatening complication characterized by severe respiratory muscle weakness, often necessitating emergent airway management and intensive care. Common triggers for MC include infections, stress, medication changes, and underlying malignancies. Standard management includes ventilatory support, immunomodulatory therapies such as plasmapheresis or intravenous immunoglobulin (IVIG), and corticosteroids. We present the case of an 85-year-old male with a history of ocular MG on pyridostigmine, Parkinson’s disease on carbidopa/levodopa, and colorectal adenocarcinoma on active chemoradiation. He presented to the emergency department with three days of worsening facial weakness, dysphagia, dysarthria, and respiratory distress. Examination revealed significant bulbar weakness, inability to clear secretions, and hypoxia despite noninvasive ventilatory support. His negative inspiratory force (NIF) deteriorated from -12 to -10, prompting emergent intubation for airway protection. Given his ongoing respiratory failure, he underwent plasmapheresis with subsequent clinical improvement. He remained intubated for four days, completed six sessions of plasmapheresis, and was transitioned to rehabilitation upon discharge. This case highlights the unpredictable nature of MC, emphasizing the need for early recognition and timely intervention in the emergency setting. It highlights the importance of multidisciplinary management, including neurology and critical care, and the role of plasmapheresis as a rapid and effective therapeutic option for severe MG exacerbations.

## Introduction

Myasthenia gravis (MG) is a chronic autoimmune neuromuscular disorder characterized by fluctuating skeletal muscle weakness due to autoantibodies targeting acetylcholine receptors (AChRs) at the neuromuscular junction [1]. The disease can range from mild ocular symptoms to severe generalized weakness, with the most critical complication being myasthenic crisis (MC), a condition marked by profound respiratory muscle weakness, leading to respiratory failure [1]. MC is a medical emergency that requires prompt recognition and intervention, including airway management and immunomodulatory therapy such as plasmapheresis or intravenous immunoglobulin (IVIG).

MC occurs in approximately 15-20% of MG patients and may be the initial presentation in some cases [2]. It is often precipitated by infections, medication changes, systemic illness, or physiological stressors such as surgery or trauma. The timely identification of MC is critical, as delayed intervention can result in significant morbidity and mortality. Airway compromise in these patients necessitates careful consideration of intubation strategies, as the use of neuromuscular blocking agents requires modification due to the underlying pathophysiology of MG.

In this case report, we present an 85-year-old male with a history of ocular MG on pyridostigmine, Parkinson’s disease, and colorectal adenocarcinoma on active chemoradiation, who developed a MC requiring emergent intubation and plasmapheresis. This case stresses the importance of early recognition and intervention in MC, highlights the challenges in airway management, and discusses the role of plasmapheresis in acute MG exacerbations.

## Case presentation

An 85-year-old male, with a past medical history of ocular MG on pyridostigmine, Parkinson’s disease on carbidopa/levodopa, and colorectal adenocarcinoma on active chemoradiation (xeloda and bevacizumab), presented to the emergency department (ED) with three days of progressively worsening facial weakness, eyelid drooping, dysarthria, dysphagia, and respiratory distress.

The patient had been diagnosed with MG several years prior and maintained on pyridostigmine 60 mg three times daily. No other medications had been recently changed in the last six months. Approximately one month prior to presentation, he developed worsening diplopia, attributed to an ocular MG exacerbation, leading to an increase in pyridostigmine dosage. As his symptoms persisted, he was started on prednisone 20 mg daily. However, three to four days before admission, he developed progressive bulbar symptoms, including difficulty swallowing, speaking, and managing oral secretions, along with increasing shortness of breath.

On the morning of the presentation, his family noted acute decompensation, including inability to clear secretions, worsening respiratory distress, and nearly complete ptosis, prompting an emergency visit. Notably, he had recently completed an outpatient antibiotic course for left lower lobe pneumonia but had no prior history of MC or intubation.

On arrival at the ED, the patient appeared to be in moderate respiratory distress, with poor air movement and weak inspiratory effort. He had copious secretions requiring frequent suctioning and was unable to fully open his eyes, close his mouth, articulate speech, or swallow secretions, consistent with significant bulbar muscle weakness. His initial vital signs were notable for a heart rate of 59 bpm, blood pressure of 154/81 mmHg, respiratory rate of 20 breaths per minute, oxygen saturation of 93% on room air, and temperature of 36.3°C.

Neurologically, he was alert and able to follow commands but demonstrated significant bulbar weakness, symmetric upper extremity weakness, and weak cough reflex. His lower extremity strength was at baseline, consistent with his Parkinson’s disease.

The patient's initial negative inspiratory force (NIF) was -12 cm H₂O, which further declined to -10 cm H₂O despite noninvasive positive pressure ventilation (NIPPV), indicating progressive respiratory muscle weakness. Arterial blood gas (ABG) analysis revealed mild respiratory alkalosis with a pH of 7.49, pCO₂ of 27.6 mmHg, and bicarbonate (HCO₃) of 21 mmol/L, suggesting increased respiratory effort (Table 1). A complete blood count (Table 2) showed mild leukocytosis (7.6 x 10³/μL), which could be indicative of a recent or ongoing infectious process. The comprehensive metabolic panel (Table 3) demonstrated normal electrolyte levels but mild renal insufficiency, with a creatinine level of 1.31 mg/dL and an estimated glomerular filtration rate (eGFR) of 53 mL/min/1.73m².

**Table 1:** Arterial blood gas

Arterial Blood Gas	Latest Reference Range & Units	Patient Values
pH Venous	7.350-7.450	7.364
pCO_2_ Venous		43.7
pO_2_ Venous		112.7
O_2_ Saturation	%	98.1
MetHemoglobin	0.0-1.5%	0.3
CarboxyHemoglobin	0.0-1.9%	1.6
Total Hemoglobin	13.2-16.6 g/dL	13.5

**Table 2:** Complete blood count (L): Data are abnormally low; (H): Data are abnormally high.

Complete Blood Count	Latest Reference Range & Units	Patient Values
Hemoglobin	13.2-16.6 g/dL	13.3
Hematocrit	38.3-48.6%	40.6
Erythrocytes	4.35-5.65x10(12)/L	3.89 (L)
MCV	78.2-97.9 fL	104.4 (H)
RBC Distrib Width	11.8-14.5%	16.2 (H)
Platelet Count	135-317x10(9)/L	262
Leukocytes	3.4-9.6x10(9)/L	7.5
Neutrophils	1.56-6.45x10(9)/L	5.62
Lymphocytes	0.95-3.07x10(9)/L	1.24
Monocytes	0.26-0.81x10(9)/L	0.57
Eosinophils	0.03-0.48x10(9)/L	0.05
Basophils	0.01-0.08x10(9)/L	0.04
Nucleated RBC	/100 WBC	0.0

**Table 3:** Basic metabolic panel, hepatic function panel, and lactate (L): Data are abnormally low; (H): Data are abnormally high.

Basic Metabolic Panel, Hepatic Function Panel, and Lactate	Latest Reference Range & Units	Patient Values
Sodium, S	135-145 mmol/L	144
Potassium, S	3.6-5.2 mmol/L	3.6
Chloride, S	98-107 mmol/L	108 (H)
Bicarbonate, S	22-29 mmol/L	24
Anion Gap	7-15	12
BUN (Blood Urea Nitrogen), S	8.0-24.0 mg/dL	20.3
Creatinine	0.74-1.35 mg/dL	1.31
Estimated GFR (eGFR)	>=60 mL/min/BSA	53 (L)
Calcium, Total, S	8.8-10.2 mg/dL	9.5
Glucose, S	70-140 mg/dL	90
Bilirubin, Total, S	0.0-1.2 mg/dL	0.9
Bilirubin, Direct, S	0.0-0.3 mg/dL	0.2
Alanine Aminotransferase (ALT), S	7-55 U/L	17
Aspartate Aminotransferase (AST), S	8-48 U/L	32
Alkaline Phosphatase, S	40-129 U/L	91
Protein, Total, S	6.3-7.9 g/dL	6.9
Albumin, S	3.5-5.0 g/dL	4.4
Lactate	0.5-2.2 mmol/L	2.0

**Figure 1:**
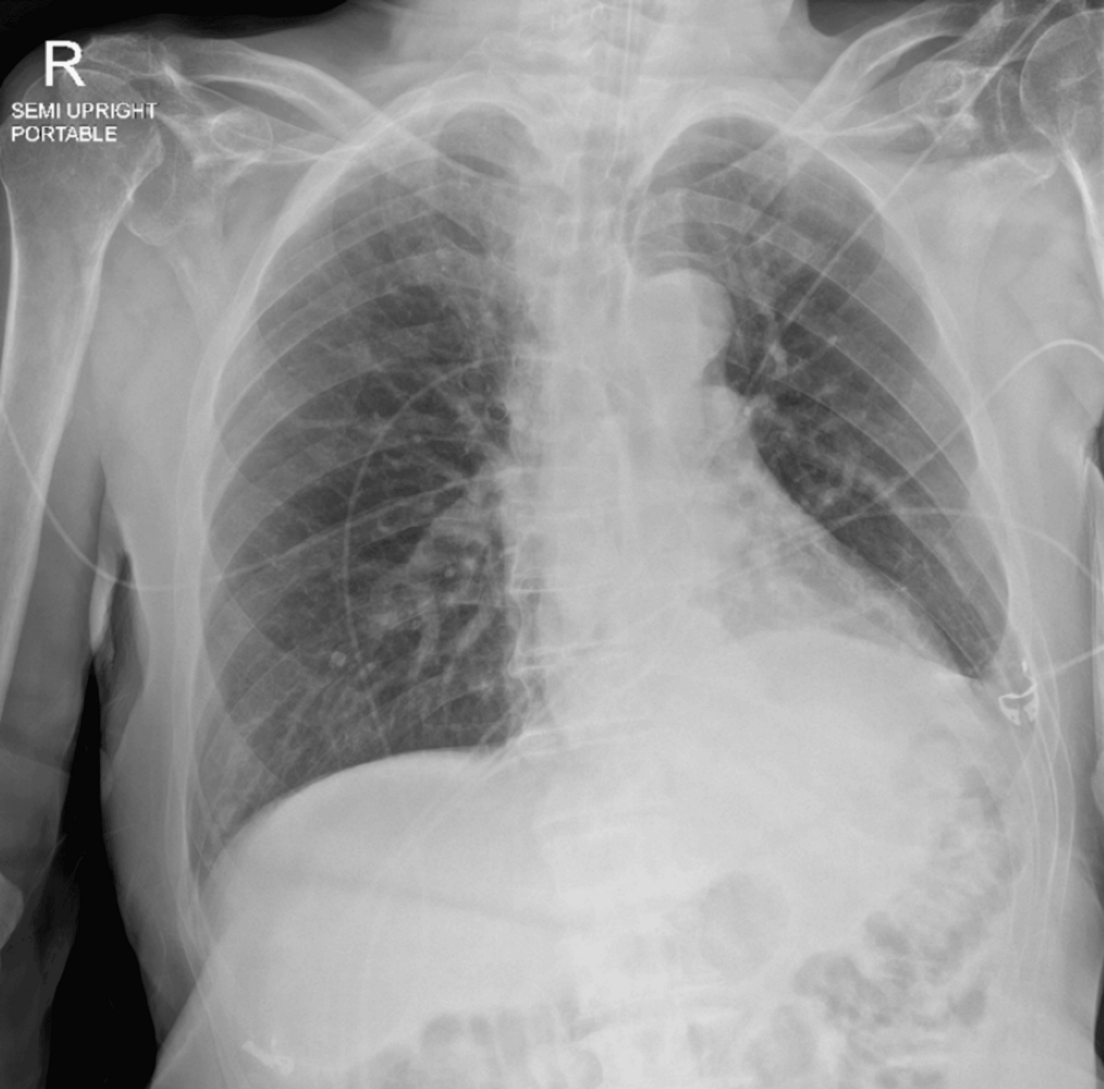
No consolidation, pleural effusion or pneumothorax. Normal cardiomediastinal silhouette

**Figure 2:**
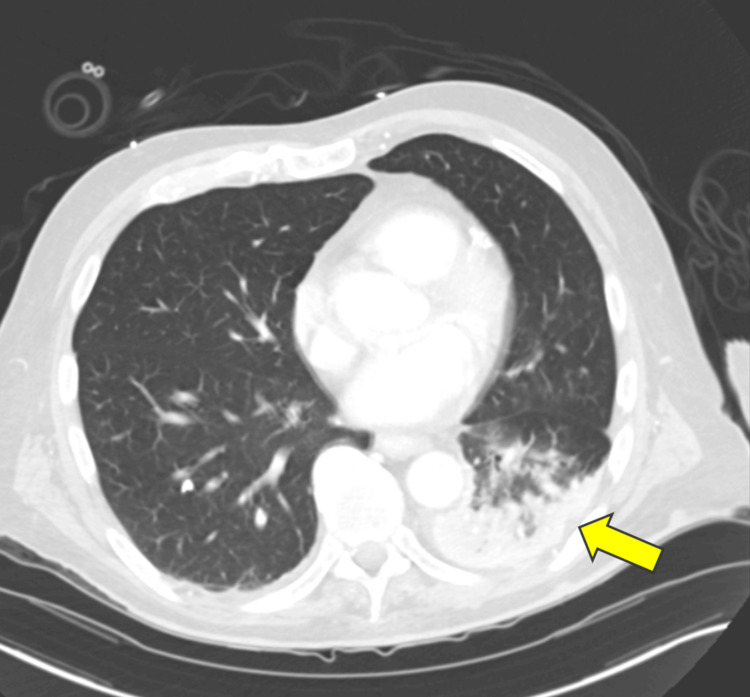
Left basilar opacities are predominantly enhancing However, there are some central regions of hypoenhancement. Overall, findings could reflect atelectasis with superimposed aspiration change (yellow arrow).

Imaging studies included a chest X-ray, which showed no evidence of consolidation, pleural effusion, or pneumothorax (Figure 1). However, a CT scan of the chest with contrast revealed left basilar opacities concerning aspiration pneumonia and multiple indeterminate pulmonary nodules (Figure 2). No evidence was seen for thymoma. A CT scan of the head was unremarkable, ruling out acute intracranial pathology as a contributing factor to his presentation.

Despite the initial trial of NIPPV (12/6 cm H₂O, 100% FiO₂), he exhibited no improvement in respiratory effort or secretion management, with worsening NIF (-10 cm H₂O). Given progressive bulbar and respiratory weakness, inability to protect his airway, and high risk for aspiration, the decision was made to proceed with emergent endotracheal intubation for airway protection and acute hypoxic respiratory failure secondary to MC.

Recognizing the neuromuscular junction dysfunction in MG, the patient was intubated using a reduced dose of (neuromuscular blocking agents due to the altered function and reduced number of acetylcholine receptors at the neuromuscular junction) rocuronium (50 mg) and standard-dose etomidate (20 mg). The procedure was successful on the first pass without complications. Post-intubation, a hemodialysis catheter was placed in the right internal jugular vein for planned plasmapheresis and possible IVIG administration. The patient was subsequently admitted to the medical intensive care unit (MICU) under neurology consultation for further management.

The patient remained intubated for four days due to increased secretions and respiratory weakness. The patient underwent six sessions of plasma exchange over a nine-day period to manage his MC, with concurrent administration of prednisone. During hospitalization, his home medication, Mestinon, was prescribed. 

Throughout the hospitalization, physical examination revealed improvements in facial and bulbar strength. NIF was monitored to assess respiratory effort and showed significant improvement. Physical and occupational therapy evaluations indicated that the patient was suitable for discharge to a skilled nursing facility (SNF) for rehabilitation.

The patient was treated for aspiration pneumonia initially with Zosyn, which was later de-escalated to ceftriaxone, completing a five-day antibiotic course. Despite treatment, concerns about aspiration persisted, as the patient failed multiple speech evaluations, including a video swallow study. After discussions with the patient and his family, a decision was made to proceed with the placement of a gastrojejunostomy tube, which was successfully completed at a later date without acute complications.

The patient was discharged in stable condition to a SNF with appropriate follow-up care in place. At discharge, the patient’s medication regimen included prednisone 20 mg daily and Mestinon 30 mg three times daily. Additionally, Bactrim was prescribed three times per week as prophylaxis for Pneumocystis jirovecii pneumonia. A neurology follow-up appointment was established to ensure continuity of care.

## Discussion

MC is a life-threatening exacerbation of MG characterized by severe respiratory muscle weakness, leading to respiratory failure. It occurs in approximately 15-20% of MG patients and may be the initial presentation in up to 20% of cases [1,2]. MC often necessitates endotracheal intubation and intensive care management [2]. Common precipitating factors include infections, medication changes, systemic illness, emotional stress, and physiological stressors such as surgery [3].

The underlying pathophysiology of MG involves an autoimmune attack on the postsynaptic membrane of the neuromuscular junction. In the majority of patients (about 85%), autoantibodies are directed against the AChRs, which play a critical role in transmitting nerve signals to muscle fibers [4]. These antibodies either block, degrade, or internalize the receptors, leading to reduced numbers of functional AChRs and inefficient neuromuscular transmission, which results in muscle weakness and fatigue [4].

In approximately 10-15% of MG patients, autoantibodies are directed against muscle-specific kinase (MuSK), a protein involved in maintaining the structure of the neuromuscular junction [5]. MuSK-positive MG tends to present with more severe bulbar symptoms and respiratory involvement compared to AChR-positive MG [6]. Other less common forms of MG include those with antibodies against lipoprotein receptor-related protein 4 (LRP4), which may also contribute to synaptic dysfunction [4].

The thymus plays a central role in the pathogenesis of MG. Up to 80% of patients with MG have thymus abnormalities [7]. In such cases, T-cell selection may be impaired due to thymic hyperplasia (seen in 75-80% of MG patients and/or thymomas (seen in 10-15% of MG patients [7]. This causes loss of or insufficient negative T-cell selection, leading to a release of autoreactive CD4 and CD8 T-cells. While it remains unclear how these T-Cells cause antibodies targeting AChR, MuSK, and/or LRP4, thymectomy, or removal of the thymus, has been shown to improve clinical outcomes, particularly in patients with thymomas [4,8].

The acute management of MC presents several challenges, including diagnostic uncertainty, atypical presentations, medication-induced exacerbations, and the need for long-term immunotherapy. In cases where bulbar symptoms predominate with minimal or no ocular involvement, distinguishing MG from bulbar amyotrophic lateral sclerosis (ALS) can be difficult. Key clinical features such as the absence of tongue atrophy, fasciculations, jaw jerk, and spastic speech can aid in differentiation. However, electrodiagnostic studies, including electromyography (EMG) and single-fiber EMG (SFEMG) of weak muscles, remain the most reliable tools for confirming an MG diagnosis [2].

Differentiating MG from bulbar ALS is crucial, particularly in patients presenting with predominant bulbar symptoms and minimal ocular involvement. While clinical features such as the absence of tongue atrophy, fasciculations, jaw jerk, and spastic speech provide initial diagnostic clues, electrodiagnostic studies remain the most reliable method for distinguishing between these conditions. Repetitive nerve stimulation (RNS) in MG typically demonstrates a decremental response (≥10% drop in compound muscle action potential (CMAP) amplitude) due to impaired neuromuscular transmission, a finding absent in ALS. In contrast, ALS primarily involves motor neuron degeneration, and while small CMAP amplitudes may be observed due to axonal loss, RNS does not show the characteristic decrement seen in MG.

Single-fiber electromyography further refines the distinction by assessing neuromuscular transmission at a more detailed level. In MG, SFEMG shows increased jitter and impulse blocking, reflecting impaired synaptic transmission, whereas, in ALS, increased jitter is also present but is due to collateral reinnervation of denervated muscle fibers rather than direct synaptic dysfunction. Additionally, fiber density is significantly increased in ALS due to chronic neurogenic remodeling, a finding absent in MG. Needle electromyography provides further confirmation, as MG typically shows normal or mildly abnormal motor unit potentials, while ALS demonstrates chronic neurogenic changes, including fibrillations, positive sharp waves, and fasciculations, indicative of ongoing motor neuron loss.

In critically ill patients experiencing acute respiratory failure, recognizing these electrodiagnostic differences is essential, as MG is a treatable autoimmune disorder, whereas ALS is a progressive neurodegenerative disease with limited treatment options. In our patient, RNS revealed a significant decremental response, and SFEMG demonstrated increased jitter without increased fiber density, supporting the diagnosis of MG over ALS.

Timely recognition and intervention in MC are critical to reducing morbidity and mortality. Clinical indicators for impending respiratory failure in MC include NIF < -30 cm H₂O, forced vital capacity (FVC) < 15 mL/kg, inability to clear secretions due to bulbar muscle weakness, and progressive hypoxia or hypercapnia. In MG patients requiring intubation, neuromuscular blocking agents (NMBAs) should be used cautiously, as they may have prolonged effects due to reduced acetylcholine receptor availability [3].

Atypical presentations, particularly in patients with anti-MuSK antibodies, can further complicate diagnosis and management. These patients may exhibit predominantly neck extensor, shoulder, and respiratory muscle weakness, or a severe oculobulbar phenotype with fixed facial weakness, tongue atrophy, and pharyngeal involvement, which can be more resistant to treatment [2]. Recognizing these variations is crucial, as delayed diagnosis and treatment can lead to prolonged respiratory failure and increased morbidity.

Patients who present with exacerbation of MG are often triggered by infections, surgical procedures, and certain medications, which can lead to rapid clinical deterioration. In our patient, recent adjustments in pyridostigmine and prednisone, ongoing chemotherapy, and a prior respiratory infection were potential contributors to his crisis. Careful medication review is essential in MG patients presenting with sudden worsening symptoms.

Our patient’s history of Parkinson’s disease likely contributed to his vulnerability to MC in several ways. Both MG and Parkinson’s disease affect neuromuscular function, albeit through distinct mechanisms: MG impairs neuromuscular transmission at the synapse, whereas Parkinson’s disease involves dopaminergic neuronal loss in the basal ganglia, leading to motor dysfunction. However, Parkinson’s disease itself may exacerbate MG-related weakness due to increased overall muscle rigidity, bradykinesia, and impaired compensatory mechanisms.

The mainstays of immunomodulatory therapy for MC include plasmapheresis and IVIG. Both therapies work by reducing circulating autoantibodies, but their mechanisms and onset of action differ. Plasmapheresis works by physically removing circulating autoantibodies, providing rapid symptom relief within days. IVIG works by modulating immune response.

IVIG has comparable efficacy to plasmapheresis in the treatment of patients with moderate-to-severe MG [9]. Patients may prefer IVIG due to a lack of requirement for dialysis catheters and its potential complications. Both treatments are well-tolerated, and the duration of effect is comparable. While long-term efficacy between both therapies was similar, plasmapheresis provided more immediate respiratory recovery, supporting its use in critically ill patients requiring mechanical ventilation [2,9]. Given our patient's severe respiratory compromise and rapid deterioration, plasmapheresis was chosen over IVIG, leading to progressive improvement over his six treatment sessions.

Long-term disease control is essential, as 33% of patients who experience MC will have a recurrence. Regular neurology follow-up is necessary to optimize maintenance immunosuppression with steroid-sparing agents such as azathioprine or mycophenolate mofetil. Additionally, while thymectomy is beneficial in younger MG patients, its role in older adults remains debated, though it may be considered in thymoma-associated MG [8].

## Conclusions

This case illustrates the importance of early recognition and aggressive intervention in MC. The patient’s rapidly worsening bulbar and respiratory muscle weakness, poor inspiratory effort, and inability to clear secretions necessitated early intubation, respiratory support, and plasmapheresis, leading to successful clinical recovery. This case is a prime example of the unpredictability of MG exacerbations, the need for multidisciplinary critical care coordination, and the role of plasmapheresis as a first-line therapy in severe MG crises requiring mechanical ventilation.
